# Circular Bioeconomy in Action: Transforming Food Wastes into Renewable Food Resources

**DOI:** 10.3390/foods13183007

**Published:** 2024-09-23

**Authors:** Priti Pal, Akhilesh Kumar Singh, Rajesh Kumar Srivastava, Saurabh Singh Rathore, Uttam Kumar Sahoo, Sanjukta Subudhi, Prakash Kumar Sarangi, Piotr Prus

**Affiliations:** 1 Shri Ramswaroop Memorial College of Engineering & Management, Tewariganj, Faizabad Road, Lucknow 226028, India; ppbst1993@gmail.com; 2 Department of Biotechnology, School of Life Sciences, Mahatma Gandhi Central University, Motihari 845401, India; akhiliit@gmail.com (A.K.S.); ssrathore@mgcub.ac.in (S.S.R.); 3 Department of Biotechnology, GIT, Gandhi Institute of Technology and Management (GITAM), Visakhapatnam 530045, India; rajeshksrivastava73@yahoo.co.in; 4 Department of Forestry, Mizoram University, Aizawl 796004, India; uttams64@gmail.com; 5 Advanced Biofuels Program, The Energy and Resources Institute, Darbari Seth Block, Habitat Place, Lodhi Road, New Delhi 110003, India; ssubudhi@teri.res.in; 6 College of Agriculture, Central Agricultural University, Imphal 795004, India; 7 Department of Agronomy, Faculty of Agriculture and Biotechnology, Bydgoszcz University of Science and Technology, Al. prof. S. Kaliskiego 7, 85-796 Bydgoszcz, Poland

**Keywords:** food waste, food additives valorization, biomass, resources

## Abstract

The growing challenge of food waste management presents a critical opportunity for advancing the circular bioeconomy, aiming to transform waste into valuable resources. This paper explores innovative strategies for converting food wastes into renewable food resources, emphasizing the integration of sustainable technologies and zero-waste principles. The main objective is to demonstrate how these approaches can contribute to a more sustainable food system by reducing environmental impacts and enhancing resource efficiency. Novel contributions of this study include the development of bioproducts from various food waste streams, highlighting the potential of underutilized resources like bread and jackfruit waste. Through case studies and experimental findings, the paper illustrates the successful application of green techniques, such as microbial fermentation and bioprocessing, in valorizing food wastes. The implications of this research extend to policy frameworks, encouraging the adoption of circular bioeconomy models that not only address waste management challenges but also foster economic growth and sustainability. These findings underscore the potential for food waste to serve as a cornerstone in the transition to a circular, regenerative economy.

## 1. Introduction

**Figure 1:**
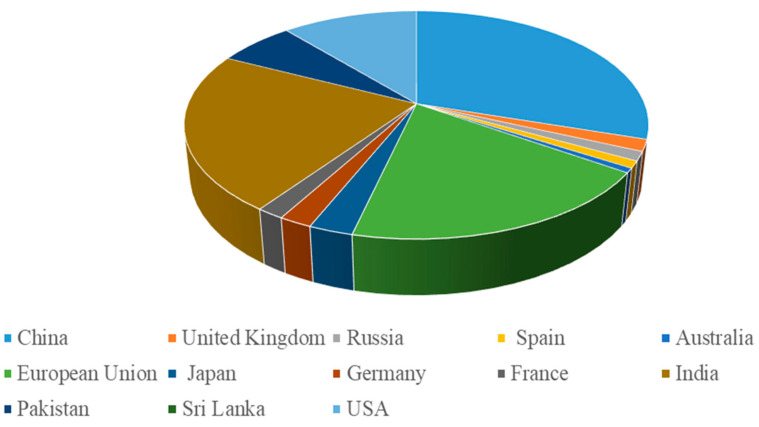
Country-wise trends in food wastes generation (Million Tonnes) around the globe.

Food waste valorization has emerged as a critical strategy for sustainable development, addressing both environmental and economic challenges. With the global food waste crisis contributing significantly to greenhouse gas emissions, innovative approaches to converting this waste into valuable products have gained momentum. Recent research highlights various advanced valorization techniques, such as the conversion of food waste into engineered biochars for CO_2_ capture, which not only reduces environmental impact but also supports a circular economy [1]. Furthermore, comprehensive reviews emphasize the potential of integrated biorefinery strategies to maximize the recovery of bioactive compounds and bioenergy from food waste, thereby turning waste into wealth and contributing to the achievement of the United Nations’ Sustainable Development Goals (SDGs) [2]. These developments underscore the growing importance of food waste valorization as a key component in global sustainability efforts [1]. In recent years, the concept of a circular bioeconomy has emerged as a transformative approach to addressing some of the most pressing environmental and economic challenges of our time. The linear model of “take–make–dispose,” which has dominated industrial practices for centuries, is increasingly recognized as unsustainable. This model leads to significant resource depletion and waste generation, contributing to environmental degradation and climate change. In contrast, a circular bioeconomy seeks to create a closed-loop system where waste is minimized, and resources are continuously reused and recycled [3]. This approach is particularly relevant in the context of food waste, which represents a substantial and growing global issue. Food waste occurs at every stage of the food supply chain, from production and processing to distribution and consumption. According to estimates by the Food and Agriculture Organization (FAO), approximately one third of all food produced for human consumption is lost or wasted, amounting to about 1.3 billion tons annually, as shown in Figure 1. 

This not only represents a colossal loss of resources such as water, land, and energy but also contributes significantly to greenhouse gas emissions with food waste accounting for roughly 8% of global emissions [4]. The concept of a circular bioeconomy offers a promising solution to this challenge by transforming food waste into valuable renewable resources. Food waste contributes to the unnecessary consumption of water, energy, and land, and when decomposed, it generates methane, which is a potent greenhouse gas that exacerbates climate change. Addressing food waste is thus critical for achieving sustainability goals and mitigating environmental impacts [5].

The circular bioeconomy provides a promising framework for transforming food waste into valuable renewable resources, reducing the environmental footprint and enhancing resource efficiency. This involves repurposing food waste into bio-based products such as chemicals, materials, and energy, or into animal feed and new food products. By converting waste into resources, the circular bioeconomy not only mitigates the adverse effects of food waste but also creates new economic opportunities and fosters innovation in sustainable practices [6].

A notable example of the circular bioeconomy in action is the transformation of coffee grounds into valuable products. Coffee is one of the most popular beverages worldwide, and the process of making coffee produces a significant amount of spent coffee grounds. Traditionally, these grounds are discarded as waste. However, recent innovations have demonstrated that spent coffee grounds can be repurposed into a variety of valuable products [7]. For instance, they can be used to produce biofuels, such as biodiesel, through a process that extracts the residual oils from the grounds. Additionally, coffee grounds can be used as a substrate for cultivating mushrooms, which not only offers a sustainable method for producing food but also contributes to the circular economy by utilizing waste as a resource [8]. The valorization of food waste involves several innovative methods and technologies. Anaerobic digestion is a well-established technology that breaks down organic waste in the absence of oxygen, producing biogas and digestate. Biogas, a renewable energy source, can be used for heating, electricity generation, or as a vehicle fuel, while digestate can be utilized as a nutrient-rich fertilizer, closing the nutrient loop [9]. Fermentation processes also play a critical role in converting food waste into valuable bio-based chemicals, materials, and even new food products. For example, fermentation can be used to produce organic acids, alcohols, and other compounds that serve as building blocks for bioplastics, biofuels, and other materials. Advanced biotechnological techniques, including genetic engineering and metabolic engineering, further enhance the efficiency and scalability of these processes, allowing for the production of high-value products from diverse waste streams [10].

Despite the promising potential of a circular bioeconomy, there are several challenges that need to be addressed. These include technical issues related to the variability and heterogeneity of food waste, which can impact the efficiency and consistency of valorization processes. Economic challenges also arise, as the financial viability of converting waste into resources depends on factors such as the market demand for bio-based products, the cost of processing technologies, and the availability of supportive policies and incentives [11]. Regulatory barriers may also hinder the adoption of innovative technologies, particularly in terms of safety and quality standards for products derived from waste. To overcome these challenges and realize the full potential of the circular bioeconomy, it is essential to foster a supportive ecosystem that encourages innovation, investment, and collaboration across sectors. This includes developing and implementing policies that promote the sustainable management of food waste, supporting research and development in waste valorization technologies, and creating market incentives for bio-based products [12]. Public awareness and education are also crucial, as they can drive consumer demand for sustainable products and practices. This paper aims to provide a comprehensive overview of the current advancements in food waste valorization, exploring the environmental, economic, and social impacts of these practices, and highlighting the critical role of innovation and policy support in achieving a circular bioeconomy. As we face growing environmental and resource challenges, the need for sustainable and innovative solutions has never been more urgent. The circular bioeconomy presents a viable pathway toward a zero-waste future, where resources are conserved, waste is minimized, and value is maximized.

## 2. Literature Review

Food waste occurs at various stages of the food supply chain, from production and processing to retail and consumption, with each stage presenting unique challenges and opportunities for waste reduction and valorization. In agricultural production, food waste begins at the source, where crops may be left unharvested or discarded due to adverse weather conditions, pest infestations, diseases, and market fluctuations [13]. Additionally, aesthetic standards set by retailers can lead to the rejection of produce that does not meet specific criteria for size, shape, or color, particularly with fruits and vegetables, where items deemed “ugly” or imperfect—such as misshapen carrots or blemished apples—are often discarded despite being nutritious and safe to eat [14]. Post-harvest handling and storage are critical stages where significant food losses occur especially in developing countries. Inadequate storage facilities, such as the lack of refrigeration, lead to the spoilage of perishable goods. For instance, in sub-Saharan Africa, the absence of proper storage and transportation infrastructure results in substantial losses of fruits and vegetables with some studies indicating that up to 50% of the produce can be lost before it reaches the market [15]. In developed countries, losses still occur due to inefficient logistics and handling practices, such as temperature fluctuations during transportation, which can cause fresh produce to spoil, leading to waste even before the goods reach consumers. The processing and packaging stages also contribute significantly to food waste due to inefficiencies in production processes, quality control measures, and packaging standards [16]. 

In the distribution and retail sectors, food waste is often driven by overstocking, improper handling, and strict aesthetic and quality standards. Retailers commonly discard food items that are close to their “sell-by” or “use-by” dates even though these items are still safe and edible. This practice is particularly prevalent with fresh produce, dairy products, and bakery items. For example, bread is frequently discarded by supermarkets once it reaches a certain age despite being perfectly suitable for consumption. Additionally, the focus on visual perfection leads to the rejection of perfectly edible but cosmetically imperfect products, exacerbating waste [17]. 

At the consumer level, food waste is often the result of over-purchasing, improper storage, and a lack of awareness about food preservation and shelf life. Households in affluent countries are particularly prone to wasting food due to buying in bulk, cooking large portions, and misunderstanding or misinterpreting food labeling. For instance, confusion between “best before” and “use by” dates often leads consumers to discard food that is still safe to eat. Bread, dairy products, and fruits are among the most commonly wasted items in households. Furthermore, cultural factors and lifestyle choices, such as a preference for variety and the convenience of ready-to-eat meals, can contribute to higher levels of food waste [18].

The environmental impact of food waste is profound. When food is wasted, all the resources used in its production, including water, land, energy, and labor, are also wasted. Moreover, food waste that ends up in landfills decomposes anaerobically, producing methane—a greenhouse gas with a global warming potential significantly higher than carbon dioxide. The carbon footprint of food waste is substantial with estimates suggesting that if food waste were a country, it would be the third-largest emitter of greenhouse gases after the United States and China. The water footprint is equally alarming; the water used to produce wasted food is equivalent to three times the volume of Lake Geneva, highlighting the inefficiencies in our current food systems [19].

Economically, the cost of food waste is immense. It includes not only the direct cost of the wasted food itself but also the cost of the resources used in its production, the expenses associated with its transportation and storage, and the cost of waste disposal. For businesses, particularly in the food retail and hospitality sectors, food waste represents a loss of potential revenue and increased operational costs. For example, restaurants that overestimate demand may end up with excess food that cannot be sold or reused, leading to waste. Globally, the economic impact of food waste is estimated at over $1 trillion annually, underscoring the scale of the issue and the potential benefits of waste reduction [20].

Socially, food waste has significant implications, particularly concerning food security and equity. While vast quantities of food are wasted, millions of people worldwide suffer from hunger and malnutrition. The redistribution of surplus food to those in need is an essential strategy for addressing this paradox. Initiatives such as food banks and community kitchens play a crucial role in capturing excess food and distributing it to vulnerable populations [21]. However, logistical challenges, food safety regulations, and a lack of awareness among potential donors can limit the effectiveness of these programs. Enhancing the capacity and efficiency of food redistribution efforts is vital for mitigating food waste’s social impact.

Therefore, the environmental, economic, and social impacts of food waste reduction are significant. Environmentally, reducing food waste leads to lower greenhouse gas emissions, less land and water use, and reduced pressure on natural ecosystems. Economically, food waste reduction can save money for businesses and consumers, create new economic opportunities, and improve resource efficiency. Socially, reducing food waste can help alleviate food insecurity, support sustainable development goals, and promote social equity. While the scale of food waste poses challenges, it also offers opportunities for innovation and positive change.

Several solutions and strategies have been proposed and implemented to address food waste. Technological innovations are among the most promising avenues for reducing waste at various stages of the food supply chain. For example, advancements in food preservation technologies, such as vacuum packaging and modified atmosphere packaging, can extend the shelf life of perishable goods, reducing spoilage [22]. Food waste represents a significant challenge in contemporary food systems both in terms of environmental impact and economic inefficiency. However, it also offers a unique opportunity for innovation and sustainability through the process of valorization—the transformation of waste materials into valuable products. Valorization not only reduces the environmental footprint of food systems but also adds economic value by creating new products from what would otherwise be discarded. Food waste valorization involves the conversion of by-products and waste materials from the food supply chain into value-added products, which can include direct human food products, animal feed, bio-based materials, and bioenergy. The concept is rooted in the principles of the circular economy, which seeks to minimize waste and make the most of resources by creating closed-loop systems. In the context of food, this means reusing and recycling materials as much as possible, reducing the need for new resources and minimizing environmental impact [23].

**Figure 2:**
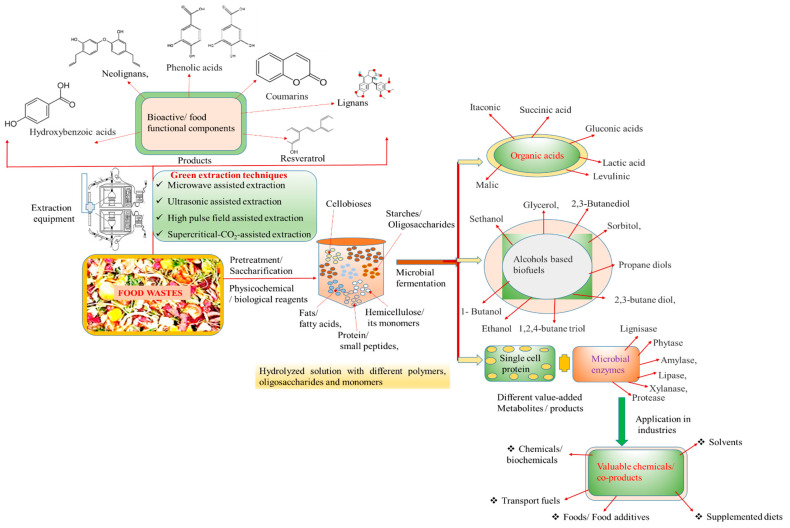
Valorization of food wastes to sustainable chemicals and food products/bioactives.

Several techniques and processes are employed in the valorization of food wastes into food products. These include mechanical processing, fermentation, enzymatic hydrolysis, extraction, and innovative biotechnological methods. Each of these processes has unique applications and potential outcomes, depending on the type of food waste and the desired end product. Mechanical processing involves the physical transformation of food waste into new products, such as grinding, drying, and pressing. For example, fruit peels and vegetable trimmings can be dried and ground into powders that are rich in fiber, vitamins, and minerals, which can then be used as ingredients in smoothies, soups, or baked goods [13]. Anaerobic digestion is a biological process that breaks down organic matter in the absence of oxygen, producing biogas and digestate, which can be used as a fertilizer. Fermentation uses microorganisms to convert organic materials into different products, such as fermented foods, bio-based chemicals, and biofuels. Enzymatic hydrolysis involves using enzymes to break down complex molecules into simpler ones, which can be used in various applications. Extraction and purification techniques isolate specific compounds from food waste for use in various food products, such as natural pigments from fruit and vegetable peels or dietary fibers from grape pomace [12]. Food waste, a significant issue in current food systems, becomes a key focus for resource recovery [24]. Food waste can be a valuable feedstock for the production of bio-based chemicals and materials. For example, citrus peel waste, rich in pectin, can be processed into pectin-based products used in the food and pharmaceutical industries. Similarly, waste oil from food processing can be converted into biodiesel, providing a renewable alternative to fossil fuels. Converting food waste into animal feed is another effective valorization strategy, reducing the environmental impact of waste disposal and decreasing the demand for conventional feed ingredients. Innovative technologies are enabling the development of new food products from food waste, such as dietary supplements and functional foods [25]. Additionally, food waste is a significant source of bioenergy, particularly through processes such as anaerobic digestion and combustion, which offer renewable and sustainable alternatives to fossil fuels. Food waste can be transformed from a problem into a valuable resource, contributing to more sustainable and resilient food systems [7,26,27]. The valorization of food wastes to sustainable chemicals and food products/bioactives is shown in Figure 2.

## 3. Methodology and Analysis

In this comprehensive review, an extensive search across renowned databases such as ISI Web of Science and Google Scholar was conducted to gather a wide range of published articles on various aspects of food waste management and valorization. The focus was particularly on exploring the nutritional properties of food waste, including the composition and types of waste generated from diverse sources. Additionally, various valorization strategies were examined, involving physical, biological, and chemical techniques, along with innovative green solvents. These advanced techniques are crucial for extracting valuable bioactive compounds such as phenolics, carotenoids, flavonoids, tannins, and antioxidants from discarded food waste [28].

The review also discusses the promising potential of microbial fermentation in transforming food waste into valuable resources. This transformative process offers opportunities to produce bioethanol, biogas, bioplastics, and other value-added products, promoting a sustainable and eco-friendly approach to waste management [22,23]. Furthermore, practical implementations of conversion technologies for food waste were explored with a thorough evaluation of their cost-effectiveness and limitations in a global context. Throughout this review, we emphasized the immense potential of food waste valorization in augmenting the share of renewable resources in the global economy while ensuring strict adherence to environmental safeguards [29]. Overall, this review provides a compelling analysis, highlighting the pivotal role of food waste valorization in fostering a circular bioeconomy and contributing to a greener, more sustainable future for our planet [30,31].

## 4. Results and Discussion

### 4.1. Compositions of Foods Waste

Food waste comprises a diverse array of nutrients, varying with the type of food. Generally, fruit and vegetable waste includes high amounts of fiber, antioxidants, and essential nutrients found in peels and rinds as well as vitamins and minerals in pulp and seeds. Overripe or spoiled produce tends to have high sugar and carbohydrate levels, which are breaking down into simpler compounds. Grain and cereal waste typically contains fiber, B vitamins, and minerals from husks and bran, while leftover cooked grains hold carbohydrates, proteins, and possibly fats [32]. Table 1 showcases how various food wastes can be transformed into valuable food products through different valorization processes. These processes not only provide new food resources but also contribute to reducing food waste and its environmental impact.

In meat and dairy products, waste such as bones and shells are rich in calcium, collagen, and other minerals, while fat and trimmings contain high lipid levels. Expired dairy products have proteins, fats, and lactose, which can be utilized in various processes like fermentation or composting. Beverage waste includes spent coffee grounds and tea leaves, which are rich in antioxidants and can be used in composting, or expired juices that are high in sugar and can be fermented into vinegar or alcohol [23].

Bread and bakery product waste features carbohydrates and proteins from stale bread, which can be repurposed as animal feed or bread crumbs. Seafood waste, including heads, shells, and bones, contains minerals and collagen and can be used in broths or animal feed, while expired seafood holds proteins and fats suitable for bioprocessing. Legumes and nuts waste includes shells and pods high in fiber, used for composting or feed, and spoiled nuts containing oils and proteins that can be processed into biodiesel or animal feed [43]. Fruit waste is rich in numerous phytochemicals, such as isoflavones, lignin, and saponins, as well as essential nutrients. However, the processing of this waste generates solid by-products, such as peels, seeds, and latex, which contribute to environmental damage. Despite this, recent research has explored the potential of food waste as eco-friendly feedstock for bioproduct synthesis. These waste materials have demonstrated promising biochemical compositions, supporting their use in renewable, bio-based product development. Further studies have focused on utilizing food waste as a sustainable source for recovering commercial pectin, biofuels, and other valuable products.

#### Various Types of Food Waste

Food waste, a significant issue in global sustainability efforts, spans a wide range of materials generated throughout the food life cycle. For instance, fruit and vegetable waste, including peels, cores, and damaged produce, often amounts to considerable quantities. Apples, for example, generate core waste, while potato peels and banana skins are commonly discarded. Research indicates that around 20–30% of fruits and vegetables are wasted in various forms, which can be redirected for composting, animal feed, or extracting bioactive compounds for health and industrial uses [44].

Grain and legume waste, such as rice husks, wheat bran, and lentil skins, represents a substantial portion of agricultural by-products. Rice husks alone can account for 15–20% of the rice harvest weight. These materials are increasingly used in animal feed, biofuels, and soil amendments, leveraging their nutritional and energy content [45]. Dairy waste, comprising expired or spoiled dairy products, whey, and cheese rinds, also poses a significant challenge. Whey, a by-product from cheese production, can constitute up to 90% of the liquid by-products in cheese making [46]. This waste is often utilized to create nutritional supplements or fermented into biogas, demonstrating its potential for resource recovery. Meat and seafood waste, including bones, trimmings, and shells, is another critical area. For example, fish bones and shrimp shells can be converted into fishmeal or chitosan. Research has shown that up to 20% of seafood processing waste can be repurposed into high-value products like chitosan, which is used in agriculture and medicine [47]. Bakery waste, such as stale bread and pastry trimmings, contributes to substantial food loss. Stale bread, which can constitute up to 5% of bakery production, is often recycled into animal feed or used in bioethanol production [48]. This approach helps mitigate waste and supports sustainable practices. Beverage waste includes spent grains from brewing, fruit pulp from juicing, and expired beverages. Spent grains, which make up about 85% of the solid by-products from brewing, are utilized as livestock feed or in biogas production [49]. Fruit pulp, accounting for 10–15% of juice production, can be transformed into jams or used as a fermentation substrate [50]. Plant waste from cultivation, such as leaves and stems left after harvesting, represents a significant portion of agricultural residue. For instance, corn stalks and tomato vines, which can make up 30–40% of the harvested plant material, are often used for composting or converted into biochar, enhancing soil health and carbon sequestration [51]. Jackfruit waste, specific to this tropical fruit, includes peels, seeds, and non-edible cores. Jackfruit seeds, which represent 15–25% of the fruit’s weight, are rich in protein and can be utilized in various applications. Research has shown that up to 60% of the jackfruit, including peels and cores, can be used to extract pectin or biofuels, highlighting the fruit’s potential for waste valorization [52].

**Figure 3:**
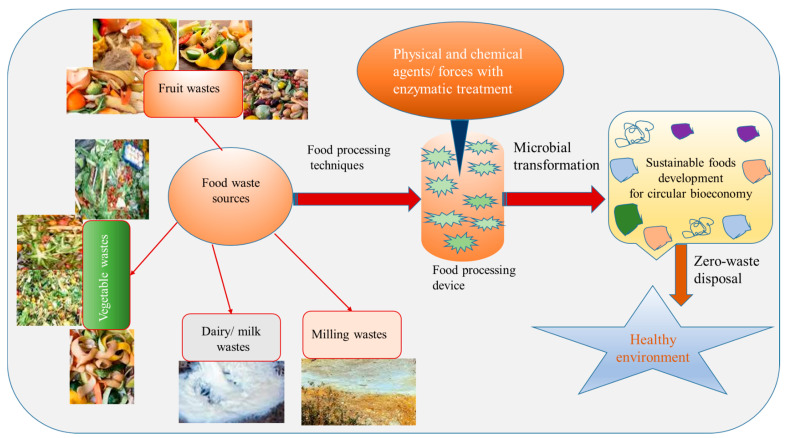
Production of food wastes from various sources and processing.

These examples underscore the diverse types of food waste and their potential for resource recovery and sustainable utilization, illustrating the importance of innovative approaches to manage and repurpose these by-products. Figure 3 illustrates the generation of food waste across different sources, highlighting the significant impact of these aesthetic rejections on overall food waste.

### 4.2. Transforming Food Wastes into Renewable Food Resources and Extraction Techniques

Utilizing waste biomass from fruits for high-value bioproducts is a promising strategy in sustainable waste management. For example, research has shown that discarded orange peels, which constitute approximately 50% of the fruit’s weight, can be valorized through various methods. These include producing biofuels, employing biorefinery processes, extracting pectin, and creating nutritious animal feed additives. In terms of biofuel production, orange peel waste has demonstrated the potential to yield up to 30% bioethanol from its cellulose content [53]. Banana waste, which includes the peel and pseudostems, also presents a significant opportunity for sustainable energy generation. With banana peels making up about 40% of the fruit’s weight, they can be utilized for biogas production through anaerobic digestion. Studies have reported that banana peel waste can generate up to 60% of biogas, demonstrating its effectiveness as a renewable energy source [54].

Similarly, apple by-products, which account for roughly 30% of the fruit’s weight, can be transformed into valuable biochemicals using eco-friendly technologies. For instance, apple pomace can be processed to recover polyphenols and pectin, contributing to the zero-waste goal. The application of these by-products in producing biochemicals has been shown to enhance the sustainability of apple waste management [55]. The expansion of significant food waste poses challenges for sustainable management. Food waste, including peels, seeds, and rinds, makes up about 60% of the fruit’s total weight [10].

**Figure 4:**
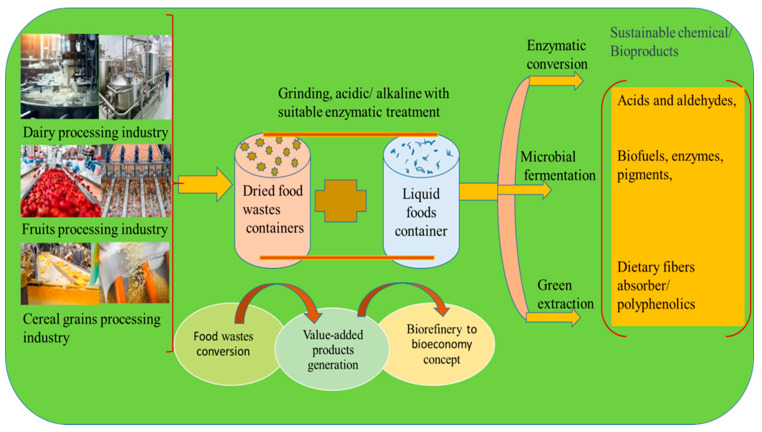
Sustainable solutions for resource recovery utilizing food wastes.

The anaerobic digestion of orange peels and apple pomace has been shown to produce significant amounts of biogas with methane yields reaching up to 300 m^3^ per ton of waste. This biogas serves as a renewable energy source, and the digestate can be used as a nutrient-rich fertilizer [11]. A recent study demonstrated the hydrolysis of banana peel waste to produce fermentable sugars, which were used to produce ethanol through microbial fermentation, yielding up to 45 g/L of ethanol. This process showcases the potential of banana peels for bioethanol production [56]. Biochar production through the pyrolysis of organic waste is a technique that enhances soil health and sequesters carbon. Research indicates that biochar made from coffee grounds and fruit peels can improve soil fertility and reduce greenhouse gas emissions. For instance, biochar derived from apple and pear waste was found to increase soil pH and nutrient content, leading to a 15% improvement in crop yields [12]. Pectin extraction from fruit peels is another valorization method. Pectin, a valuable polysaccharide, can be extracted and used as a gelling agent in the food industry. Recent research has achieved the efficient extraction of pectin from citrus peels with yields of up to 20% by weight [57]. Microbial bioprocessing involves using microorganisms to convert food waste into high-value bioproducts such as enzymes, proteins, and bioactive compounds. Recent studies have explored the use of fungi and bacteria for this purpose. For example, the filamentous fungus *Aspergillus niger* has been used to produce cellulase and xylanase enzymes from agricultural residues like wheat straw and corn cobs. These enzymes are valuable in industrial processes, including biofuel production and paper processing [58]. Upcycling food waste into functional foods with enhanced nutritional properties is another effective strategy. Recent research has shown that spent coffee grounds can be upcycled into high-protein flour, which can be used in baked goods. This flour contains significant levels of protein and dietary fiber, making it a nutritious addition to various food products [59]. These valorization techniques not only help reduce waste but also offer sustainable solutions for resource recovery and environmental protection (Figure 4).

#### Advanced and Green Extraction Techniques for Recovering Bioactives from Food Waste

Researchers have explored various novel extraction techniques for valorizing food waste, aiming to derive bioactive compounds for use in food, pharmaceutical, cosmetic, and healthcare industries. These techniques include enzyme-assisted extraction, microwave-assisted extraction, ultrasound-assisted extraction, high-hydrostatic pressure extraction, pulsed-electric field extraction, and supercritical fluid extraction. These advanced green methods are designed to achieve zero waste, contributing to sustainable energy conservation and more efficient resource use [60]. Recent studies have highlighted the potential of microbial fermentation to produce biofuels like bioethanol and biogas. For example, *Saccharomyces cerevisiae* has been used to produce bioethanol at concentrations of 11–13% and biogas through anaerobic digestion processes [61]. In addition to biofuels, microbial fermentation is also effective in generating other valuable products, including bioactive compounds. Researchers are continuously working on developing efficient techniques that avoid solvent contamination with enzyme-assisted and pressurized liquid-assisted extractions showing promise for recovering valuable compounds from jackfruit waste and other sources [62].

Microwave-assisted extraction and ultrasound-assisted extraction have proven useful for extracting specific compounds like pectin polysaccharides and antioxidant phenolics, respectively [63]. Pulsed electric field-assisted and supercritical fluid-assisted techniques have also been explored for their ability to recover bioactive compounds at high yields. The effectiveness of these green extraction methods often depends on the properties of the source material, including its chemical structure, and various process parameters such as solvent, pressure, time, and temperature [64].

Enzyme-assisted extraction (EAE) involves factors such as enzyme concentration, particle size, and hydrolysis time, which can influence the yield of bioactive compounds. For example, carotenoids have been extracted from pumpkin waste, and anthocyanins have been extracted from Crocus sativus and grape fruit waste using EAE. The process has also been successful in extracting phenolics from grape seeds and antioxidant phenols from apple pomace, demonstrating its effectiveness for various types of food waste [65].

Ultrasound-assisted extraction (UAE) offers benefits like higher yield and quality with minimal environmental impact. UAE uses sound waves in the frequency range of 20–2000 kHz to create cavitation effects that enhance mass transfer and compound extraction. The process improves solvent accessibility and accelerates the disintegration of cell walls, leading to better extraction efficiency [66]. 

Recent advancements in green extraction techniques for bioactive compounds from food waste have demonstrated significant improvements over traditional methods. These techniques include Ultrasound-Assisted Extraction (UAE), Pulse Electric Field-Assisted Extraction (PEF-AE), Microwave-Assisted Extraction (MAE), and Supercritical Fluid Extraction (SCFE).

Ultrasound-Assisted Extraction (UAE): UAE has shown considerable success in enhancing the yield of bioactive compounds. For example, tannin extraction from Avaram shell using UAE at 100 W power resulted in a 160% increase in yield. This improvement is attributed to enhanced mass transfer and the leaching of tannins due to ultrasonic waves. UAE has also been effective in extracting phenolic acids like caffeic acid (~64.3 µg/g), ferulic acid (~1513 µg/g), and p-coumaric acid (~140 µg/g), outperforming conventional maceration methods [67]. However, prolonged extraction times and high temperatures can negatively impact the yield of phenolic compounds from sources like citrus peels. Comparative studies indicate that UAE significantly reduces extraction time, achieving results in just 1 h compared to 72 h with maceration for phenolic compounds from Punica granatum fruits [67,68].

Pulse Electric Field-Assisted Extraction (PEF-AE): PEF-AE uses high-voltage pulses applied between electrodes to enhance the extraction of bioactive compounds. This non-thermal process disrupts cell structures, improving the yield of compounds by allowing molecules to separate more efficiently. PEF-AE can be applied in both batch and continuous modes [69]. Factors such as the field strength, energy, pulse number, and temperature influence the extraction efficiency. Studies have demonstrated that PEF-AE improves anthocyanin and phenolic compound yields, particularly in wine making, where it reduces maceration time and enhances the quality of the wine [69]. The technique also shows promise in increasing yields from waste materials like jackfruit.

Microwave-Assisted Extraction (MAE): MAE employs microwave energy to heat solvents, increasing the efficiency of bioactive compound extraction. For instance, MAE has achieved an 83% yield of flavonoids from *Terminalia bellerica* compared to 64% with conventional methods [70]. This technique has also successfully extracted hesperidin from Citrus unshiu fruits, although high temperatures can reduce yields by interfering with compound solubility. MAE has demonstrated superior results in extracting phenolic compounds from chokeberries and silibinin from Silybum marianum waste, showing up to 74% recovery, which is higher than traditional extraction methods. Factors influencing MAE include temperature, solvent nature, and microwave power [70,71].

Supercritical Fluid Extraction (SCFE): SCFE uses solvents above their critical point, where they exhibit properties of both liquids and gases, to extract bioactive compounds efficiently. Supercritical CO_2_ is commonly used with ethanol as a modifier. SCFE has been effective in extracting naringin from citrus paradise and phenolic compounds from rice wine lees with reduced extraction times compared to traditional methods (1 h vs. 6 h) [72]. Despite its advantages, SCFE faces challenges such as high costs, complex equipment, and issues with solvent polarity. Polar solvents like methanol or ethanol can improve extraction efficiency by enhancing the solvent properties and minimizing interactions between analytes and matrices [72,73]. Overall, these advanced extraction techniques offer enhanced efficiency, higher yields, and reduced processing times compared to conventional methods, contributing to a more sustainable and effective valorization of food waste.

### 4.3. Circular Bioeconomy in Action: Transforming Food Wastes into Value-Added Products

The circular economy revolves around transitioning from the traditional linear economy, characterized by the “take–make–dispose” model, to one that emphasizes sustainability, resource efficiency, and waste minimization. In a linear economy, raw materials are extracted, transformed into products, and discarded after use, resulting in significant resource depletion and environmental degradation. On the other hand, the circular economy focuses on creating closed-loop systems that reduce resource input, extend product life cycles, and recover and regenerate materials at the end of their service life. The two major principles of the circular economy include (1) product design for longevity and reusability and (2) waste as a resource systems thinking. This perspective promotes the development of integrated and resilient economic models.

The circular bioeconomy is a subset of the circular economy specifically focusing on the sustainable utilization of biological resources. It integrates the principles of circularity with the bioeconomy, which is an economic system that emphasizes the use of renewable biological resources—such as biomass, plants, animals, and microorganisms. This approach not only enhances resource efficiency but also aims to mitigate climate change, biodiversity loss, and environmental degradation. It is centered on maximizing resource efficiency by converting biowaste into valuable products, extending the materials’ lifespan and minimizing environmental impact. At its core, this concept envisions a closed-loop system where waste is not discarded but repurposed into high-value products, transforming biowaste management from disposal to resource recovery and assuming a zero-waste concept. This shift enables numerous innovative applications that align with sustainability goals, reduce waste, and conserve natural resources. This regenerative approach facilitates the extraction of valuable bioactive compounds—such as antioxidants, phenolic acids, and dietary fibers—from food waste, which can be used in the pharmaceutical, cosmetic, and functional food sectors. Recovering these compounds adds economic value and supports human health and well-being. Additionally, transforming food waste into animal feed or nutrient-rich compost helps close the loop by returning essential nutrients to the soil, promoting sustainable agricultural practices. This integrated strategy ensures that food waste is continually cycled back into the economy, reducing the need for new resources and enhancing environmental stewardship. In light of these principles, this section examines innovative strategies for converting food waste into high-value products. These include the production of biofuels for renewable energy, biopolymers as sustainable alternatives to conventional plastics, and bioactive compounds for pharmaceutical and cosmetic applications. It also covers the creation of functional foods enriched with nutrients, the production of animal feed from food scraps, the development of compost to improve soil health, and the extraction of natural additives like colorants and flavors. Additionally, bio-based catalysts from food wastes and their applications are critically described and analyzed. These diverse approaches illustrate the significant economic, environmental, and social benefits of transforming food waste.

#### 4.3.1. Biofuels from Food Waste

Converting food waste into biofuels, such as bioethanol and biogas, represents a promising strategy for waste valorization within the circular bioeconomy. This approach leverages the organic content of food waste to create renewable energy sources, effectively turning waste into a valuable resource. Recent studies have highlighted that food waste, such as bread waste, can be fermented using specific yeast strains like Saccharomyces cerevisiae, resulting in bioethanol yields ranging from 11% to 13% [74]. This fermentation process efficiently transforms carbohydrates in food waste into ethanol, which can be used as a sustainable fuel alternative, thus reducing dependency on conventional fossil fuels. In addition to bioethanol, anaerobic digestion is another effective method for converting food waste into bioenergy. This process involves the microbial breakdown of organic matter in the absence of oxygen, producing biogas with a methane content of up to 65% [74]. The high methane concentration in the biogas makes it an excellent source for energy production, and it is suitable for generating electricity and heat or as a vehicle fuel after purification. These biofuel production methods provide multiple benefits beyond energy generation. They significantly reduce the volume of waste sent to landfills, decreasing methane emissions associated with waste decomposition under anaerobic conditions. Moreover, using food waste for biofuel production aligns with the circular bioeconomy’s objectives by reducing greenhouse gas emissions, lowering the reliance on non-renewable energy sources, and contributing to a more sustainable and resilient energy infrastructure. By utilizing food waste for biofuel production, we address waste management challenges and promote the development of renewable energy systems that support environmental and economic sustainability. This dual benefit makes converting food waste into biofuels a key component of circular bioeconomic strategies to achieve long-term ecological balance and resource efficiency.

#### 4.3.2. Biopolymers and Biodegradable Plastics

Biopolymers derived from food waste are emerging as environmentally friendly alternatives to traditional petroleum-based plastics. One notable example is pectin, which is a valuable biopolymer extracted from citrus peels and apple pomace. Pectin has wide applications in the food and pharmaceutical industries due to its gelling, thickening, and stabilizing properties. Recent advancements in extraction technology, such as microwave-assisted extraction (MAE), have significantly improved the efficiency of pectin recovery. MAE can achieve a pectin yield of up to 83%, which is a substantial increase compared to traditional extraction methods [75]. This enhanced yield makes the process more economically viable and maximizes the value derived from food waste. In addition to pectin, there is growing interest in using food waste to produce biodegradable plastics. These plastics are designed to break down more quickly and safely than conventional plastics, reducing their long-term environmental impact. By converting food waste into biodegradable plastics, this approach supports the principles of a circular bioeconomy by diverting waste from landfills, reducing dependence on fossil fuels, and mitigating plastic pollution. The development and commercialization of biodegradable plastics from food waste present new market opportunities and contribute to more sustainable packaging solutions. Using food waste to produce biopolymers and biodegradable plastics aligns with circular economy principles by transforming waste materials into valuable products. This not only helps reduce synthetic plastics’ environmental footprint but also fosters innovation and opens up new avenues for market development.

#### 4.3.3. Recovery of Bioactive Compounds

The recovery of bioactive compounds from food waste is a significant and promising application within the circular bioeconomy, offering substantial benefits for sustainability and resource utilization. Bioactive compounds, particularly phenolic acids, are renowned for their antioxidant properties and their potential uses in pharmaceutical and cosmetic applications. These compounds can provide valuable health benefits and contribute to various industries, enhancing the overall value derived from food waste. Recent technological advancements have greatly improved the efficiency of extracting these bioactive compounds from food waste. Among these advancements, enzyme-assisted extraction (EAE) and ultrasound-assisted extraction (UAE) have emerged as effective techniques. EAE utilizes specific enzymes to break down the cellular structure of food waste, releasing bioactive compounds more efficiently. On the other hand, UAE employs high-frequency sound waves to create microbubbles in the extraction solvent, which helps in breaking down the cell walls and enhancing the release of target compounds. For instance, UAE has been successfully applied to extract caffeic acid and ferulic acid from food waste, achieving yields of approximately 64.3 µg/g and 1513 µg/g, respectively [76,77]. Caffeic acid and ferulic acid are both valuable phenolic acids with significant antioxidant activities, which are helpful in preventing oxidative damage in various products. The high yields obtained through UAE demonstrate its effectiveness in maximizing the recovery of these bioactive compounds from food waste, thus making the process more efficient and economically viable. By enhancing the extraction of bioactive compounds, these technologies contribute to the sustainability of the food processing industry. They enable the conversion of food waste into high-value products, reducing waste and creating new economic opportunities. This approach supports the circular bioeconomy by transforming discarded materials into valuable resources while also driving innovation in resource recovery.

#### 4.3.4. Development of Functional Foods

The development of functional foods from food waste presents a significant opportunity to enhance nutritional profiles and address health concerns while simultaneously reducing waste. Food waste, particularly from fruit and vegetable peels, can be repurposed to extract valuable dietary fibers and antioxidants. These components can be incorporated into new food products, improving their health benefits and contributing to a more sustainable food system. Recent research has demonstrated the potential of utilizing jackfruit waste as a rich source of antioxidants for functional food development. Jackfruit waste, which typically includes peels and seeds, has been found to contain high antioxidants capable of combating oxidative stress. Antioxidants such as polyphenols and flavonoids extracted from jackfruit waste can enhance the nutritional value of food products, potentially reducing the risk of chronic diseases and promoting overall health [78]. By integrating these antioxidant-rich extracts into food products, manufacturers can address the growing consumer demand for healthier and more sustainable food options. This approach adds value to what would otherwise be waste and aligns with broader health and environmental goals. The valorization of food waste through functional food development supports a circular bioeconomy by minimizing waste generation, conserving resources, and contributing to creating nutritious products that benefit public health.

#### 4.3.5. Animal Feed Production

Transforming food waste into animal feed provides a sustainable alternative to traditional feed sources, offering several environmental and economic benefits. This process involves repurposing various food scraps and by-products, such as brewer’s spent grains and fruit pomace, into nutrient-rich feed for livestock. These by-products, often considered waste in food production, can be processed and utilized as high-quality feed ingredients. Research has demonstrated that incorporating such food waste-derived feeds can significantly improve animal health and growth rates, making them a viable alternative to conventional feed ingredients [79]. By using food waste in animal feed production, we efficiently use resources and address several key issues associated with traditional feed sources. First, this practice reduces the reliance on primary feed crops, which can have substantial environmental impacts due to land use and resource consumption. Second, it minimizes the environmental footprint of animal agriculture by lowering feed-related greenhouse gas emissions. For instance, repurposing food scraps helps avoid the methane emissions from decomposing organic waste in landfills. Additionally, this approach contributes to enhanced food security by providing a cost-effective feed option. By converting food waste into valuable animal feed, the overall cost of feed production is reduced, which can be particularly beneficial for farmers and livestock producers. This aligns with the principles of the circular bioeconomy by closing the loop on food waste and transforming it into a resource that supports agricultural sustainability and reduces waste.

#### 4.3.6. Composting and Soil Enrichment

Composting food waste is an essential practice within the circular bioeconomy, as it transforms organic waste into nutrient-rich compost that enhances soil health and fertility. This process addresses multiple environmental concerns: it reduces the volume of waste sent to landfills, decreases emissions of methane, which is a potent greenhouse gas released from decomposing organic matter, and returns valuable nutrients to the soil, supporting sustainable agricultural practices. Recent studies have shown that compost produced from food waste can significantly improve soil health. Composting food waste enriches the soil with essential nutrients and organic matter, which enhances soil structure, water retention, and aeration. These improvements facilitate better plant growth and development by providing the necessary nutrients and creating a more conducive growing environment. Furthermore, using compost reduces the reliance on synthetic fertilizers, which can have adverse environmental impacts, such as waterway pollution and soil degradation. For example, research indicates that compost made from food waste can positively impact soil nutrient content and structure, leading to improved crop yields and reduced need for chemical inputs [80]. This practice supports agricultural productivity and contributes to the sustainable management of natural resources by closing the nutrient loop—where waste is effectively recycled back into the soil.

#### 4.3.7. Production of Natural Colorants and Flavors

Food waste can be a valuable resource for producing natural colorants and flavors, offering a sustainable alternative to synthetic additives. Extracting pigments from food waste, such as beetroot and carrot peels, provides a means to replace artificial dyes with natural options in food products. This shift addresses consumer demand for natural ingredients and leverages materials that would otherwise be discarded. Recent advancements in extraction technologies have significantly enhanced the efficiency of obtaining these natural colorants. Techniques such as optimized solvent extraction, supercritical fluid extraction, and enzyme-assisted extraction have improved the yield and quality of pigments derived from food waste [79]. These innovations make natural colorants more commercially viable by reducing production costs and increasing their application range. The use of natural colorants extracted from food waste offers several benefits. Environmentally, it reduces reliance on synthetic dyes, which can have adverse ecological effects during production and disposal. Repurposing food waste mitigates waste generation and contributes to a more circular economy. Additionally, the application of natural colorants aligns with growing consumer preferences for products free from artificial additives, enhancing the market appeal of food items. Overall, producing natural colorants and flavors from food waste exemplifies how innovative technologies can transform waste into valuable resources, reducing environmental impact and adding economic value to otherwise discarded materials [79].

#### 4.3.8. Production of Bio-Based Renewable Catalysts

**Figure 5:**
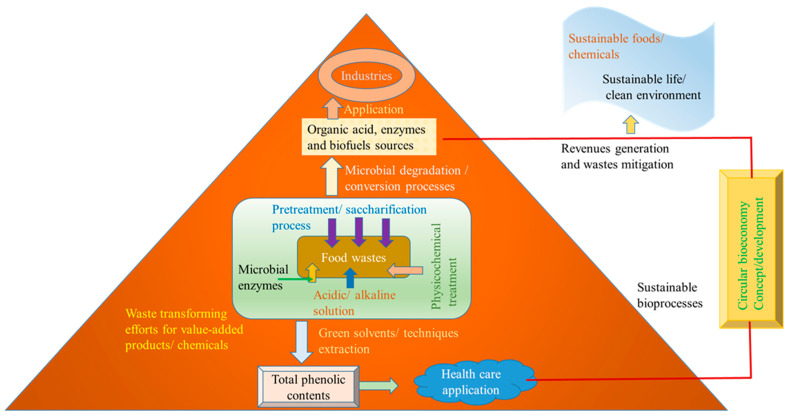
Food wastes valorization strategies for value-added production with biorefinery and bioeconomy concept.

Bio-based heterogeneous catalysts have gained promising potential as they originate from waste biomass and simultaneously also aid in the conversion of waste biomass to bioenergy and other high-value products. This focuses perfectly on circular bioeconomy principles, where waste is not simply disposed of but rather utilized to reduce environmental impacts [6]. Currently, the literature lacks significant research on the production and application of food waste into biomass catalysts, presenting a unique challenge for researchers. This scarcity of information has driven efforts to explore food waste as a potential catalyst in biodiesel production, aiming to bridge the knowledge gap in catalyst development. By focusing on food waste—an abundantly available agricultural waste—the research seeks to transform it into a valuable resource, specifically catalysts that effectively convert waste cooking oil (WCO) into biodiesel. Notably, using potassium oxide (K_2_O) derived from food waste ash as a cost-effective solid catalyst for biodiesel production has proven feasible [26]. Leveraging food waste as a solid catalyst offers several advantages, including environmental benefits by repurposing waste material, thus contributing to waste reduction and promoting sustainability in biodiesel production. The catalyst is renewable, reusable, recyclable, non-hazardous, and environmentally friendly with wide-ranging applications. The United Nations has made significant strides toward securing a sustainable future by 2030 with a strong focus on the Sustainable Development Goals (SDGs). Specifically, SDG 7 emphasizes the sustainable use of bioresources to increase the share of renewable energy in the global energy mix while also ensuring that sustainable energy services are accessible to all nations [27]. Thus, in this context, food wastes are valuable and promising resources that acting as catalysts, converting the former into valuable resources. Hence, circular bioeconomy strategies allow for the transformation of food waste into sustainable economic products, contributing to a more sustainable future (Figure 5).

## 5. Challenges, and Future Research Orientations

Valorization techniques play a crucial role in converting food wastes into valuable resources, contributing significantly to the principles of a circular bioeconomy. Recent advancements highlight several advantages of these techniques. For instance, the application of advanced methods like supercritical fluid extraction (SFE) and microwave-assisted extraction (MAE) has demonstrated substantial improvements in efficiency and sustainability. SFE, using CO_2_ as a solvent, has been particularly noted for its high yield and reduced environmental impact. Studies show that SFE with ethanol as a modifier can achieve up to a 43% yield of phenolic compounds from rice wine lees, demonstrating its effectiveness in minimizing solvent use and processing time [80,81]. Similarly, MAE has proven effective in extracting high yields of flavonoids and phenolic compounds with recent data indicating up to an 83% yield for flavonoids from *Terminalia bellerica*, showcasing its superior performance over traditional methods [70,71].

However, these advanced valorization techniques have limitations. The high initial costs associated with the setup and operation of SFE and PEF-AE equipment can be a significant barrier for widespread adoption, particularly for small-scale operations. For example, PEF-AE, despite its efficiency in enhancing the extraction yields of bioactive compounds, involves expensive infrastructure and the complex optimization of parameters such as field strength and pulse duration [63,64]. Additionally, these techniques often require the precise control and optimization of process parameters, adding to their operational complexity. MAE, for instance, demands a careful management of microwave power to avoid the overheating and degradation of sensitive compounds, further complicating its application [73,74].

Moreover, scalability issues can arise when transitioning from laboratory-scale to industrial-scale operations. The consistency and reproducibility of advanced techniques like SFE are critical challenges that can impact their commercial viability. This issue is particularly evident in the case of SFE, where maintaining process stability on a larger scale remains a significant challenge [80,82]. Despite their advantages, advanced techniques also have resource and energy requirements that must be managed carefully. While these are generally more eco-friendly than traditional methods, the use of solvents and energy consumption in processes like SFE still pose environmental concerns if not properly addressed [79,80]. The sustainability of valorization processes is critical for effective waste management, encompassing reducing, reusing, and recycling to support a circular bioeconomy. The environmental, ecological, and social impacts of end-use products derived from fruit waste must also be considered with green technologies generally offering more eco-friendly alternatives compared to conventional methods [83].

To maximize the potential of food waste, further research is needed on advanced pre-treatment and bioprocessing methods, including physico-chemical, biological, and innovative green approaches. The continued exploration of genetically and biotechnologically advanced methods could enable the complete utilization of food waste, promoting the development of a wide array of value-added products. Advances in technology, equipment, and methodologies are essential to enhance the efficiency of biorefineries and expand the production of renewable resources from food wastes. Overall, while the recent advancements in valorization techniques offer promising benefits for transforming food wastes into renewable resources, careful consideration of their limitations and drawbacks is essential for their successful implementation in the circular bioeconomy. Balancing the advantages of efficiency and sustainability with the challenges of cost, scalability, and environmental impact remains key to optimizing these technologies.

Food waste is a significant global issue with millions of tons discarded annually. This waste not only represents a loss of resources but also contributes to environmental problems such as greenhouse gas emissions from landfills. However, food waste is rich in organic matter and various functional compounds, making it a potential resource for pollution management. Transforming food waste into bio-absorbents offers a dual benefit: reducing the environmental impact of waste while providing a sustainable solution for pollution remediation. The key to this transformation lies in understanding the properties of different types of food waste and their suitability for absorbing specific pollutants [7,84].

Bio-absorption is the process by which natural materials absorb and retain contaminants, removing them from water, soil, or air. The effectiveness of bio-absorbents depends on their physical and chemical properties, including surface area, porosity, and the presence of functional groups that interact with pollutants. Food waste, with its diverse composition, offers various bio-absorption mechanisms. For instance, the high cellulose, hemicellulose, and lignin content in fruit and vegetable waste provides a porous structure that can trap pollutants. Additionally, the presence of proteins, lipids, and other organic compounds in food waste can facilitate the binding of heavy metals, dyes, and other contaminants [85]. Different types of food waste have been studied for their bio-absorbent properties, each offering unique advantages. Fruit and vegetable waste, for example, is rich in fiber and organic acids and has shown promise in absorbing heavy metals and dyes. A study demonstrated that citrus peels, which contain pectin, a polysaccharide, can chelate metal ions, making them effective in removing heavy metals like lead and cadmium from water [86]. Bread waste, due to its starch content and porous structure, can act as an absorbent for oils and organic pollutants. The amylase in bread waste can interact with specific contaminants, enhancing its absorption capacity. For instance, research found that bread waste could effectively remove up to 75% of oil residues from wastewater, making it a viable option for treating industrial effluents [87]. Cereal waste, with its high carbohydrate content, is suitable for absorbing organic pollutants. Moreover, the protein content in cereal waste can bind with heavy metals, facilitating their removal from contaminated water. Researcher highlighted that rice husk, a common cereal waste, could remove up to 90% of arsenic from polluted water, demonstrating its potential as a bio-absorbent [88]. Jackfruit waste, particularly the fibrous structure combined with its high lignin and cellulose content, is effective for absorbing organic dyes and heavy metals. In regions where jackfruit is abundant, such as southeast Asia, utilizing this waste for pollution management could offer a localized and sustainable solution. Research found that jackfruit peel could remove up to 80% of methylene blue dye from wastewater, making it a promising material for dye-contaminated effluents [89]. Spent coffee grounds are another promising food waste material due to their high surface area and the presence of active compounds like caffeine and polyphenols, which can bind with heavy metals and organic pollutants. For example, research showed that spent coffee grounds could effectively remove up to 70% of lead and 65% of chromium from contaminated water, highlighting their potential as a cost-effective and sustainable bio-absorbent [90]. Integrating food waste-based bio-absorbents into pollution management practices aligns with several principles of the circular bioeconomy. By utilizing food waste, we can reduce the demand for virgin materials, such as synthetic absorbents, which often require energy-intensive production processes [91]. Additionally, transforming food waste into bio-absorbents prevents it from being landfilled or incinerated, reducing greenhouse gas emissions and other environmental impacts. This approach also adds value to what would otherwise be considered waste, creating economic opportunities, particularly in regions where food waste is abundant. While the potential of food waste as a bio-absorbent is promising, several challenges need to be addressed to fully realize its benefits. Developing large-scale processes for converting food waste into bio-absorbents remains a challenge [92]. Research is needed to optimize production methods, improve efficiency, and reduce costs. Different types of food waste have varying absorption capacities for different pollutants, so more research is needed to identify the best matches between specific food waste materials and contaminants. Additionally, the use of food waste in pollution management must meet regulatory standards to ensure that it does not introduce new contaminants into the environment. Safety protocols and guidelines need to be established for the use of food waste-based bio-absorbents. For food waste-based bio-absorbents to be widely adopted, public awareness and acceptance are crucial. Education and outreach efforts can help promote the benefits of this sustainable approach to pollution management. For instance, community-level initiatives that demonstrate the effectiveness of food waste-based bio-absorbents in local water treatment projects could play a key role in gaining public trust and encouraging wider adoption. The utilization of food waste with the help of innovative approaches and scientific methods for valuable sustainable products is focused as far as environmental, food, and energy sectors are considered [93,94,95]. Zero-waste approaches are to be adapted for food wastes so that environmental sustainability can be maintained.

## 6. Conclusions

The exploration of circular bioeconomy principles applied to food waste management has demonstrated significant potential for transforming food wastes into renewable food resources. This approach not only addresses the critical issue of food waste reduction but also contributes to sustainable development by closing the loop on resource use. By converting food waste into valuable products such as bio-based materials, biochemicals, and bioenergy, we can reduce reliance on non-renewable resources, decrease environmental impacts, and foster economic growth. This review also highlights several strategies for developing sustainable fuels while maintaining a clean environment. It provides insights into the diverse nature of food waste and its nutrient content, which has been extensively analyzed and studied. Furthermore, various valorization techniques, such as green extraction, bioactive material preparation, and microbial fermentation, are discussed in greater depth along with the mechanisms for generating and recovering nutrients and bioproducts. These conversion approaches have proven effective in producing substantial amounts of bioproducts from food waste, contributing to environmental cleanliness and safety. This strategy aligns with the circular bioeconomy goal of achieving zero waste through comprehensive conversion processes. The integration of food waste into the circular bioeconomy framework offers numerous environmental, economic, and social benefits. Environmentally, it helps mitigate climate change by reducing greenhouse gas emissions associated with food waste decomposition in landfills. Economically, it creates new value chains and market opportunities, particularly for local communities and industries. Socially, it contributes to food security by enabling the recovery of nutrients and other valuable components from food waste, which can be reintegrated into the food supply chain.

There are several challenges and areas that require further research to fully realize the benefits of transforming food wastes into renewable food resources. The scalability of food waste valorization processes is one of the primary challenges. Although numerous small-scale and pilot projects have shown promising results, there is a need for more extensive research and development to optimize these processes for large-scale application. This includes improving the efficiency and cost-effectiveness of food waste conversion technologies and developing robust supply chains for collecting and processing food waste. Another critical area for future research is the development of innovative technologies and processes that can enhance the conversion of food waste into high-value products. This includes exploring new methods for extracting bioactive compounds, improving fermentation processes for bioenergy production, and developing advanced materials from food waste. Additionally, research should focus on the potential of using food waste in precision agriculture, where nutrients recovered from food waste can be utilized to improve soil health and crop yields. The diversity of food waste types presents another challenge, as different types of food waste require specific treatment processes to maximize their valorization potential. Future research should aim to develop tailored approaches for different food waste streams, taking into account regional variations in food waste composition and availability. Moreover, there is a need for more comprehensive life cycle assessments (LCAs) to evaluate the environmental impacts of food waste valorization processes and ensure that they align with sustainability goals. Regulatory frameworks and policies play a crucial role in facilitating the adoption of circular bioeconomy practices. Therefore, future research should also focus on identifying and addressing regulatory barriers that hinder the transformation of food waste into renewable resources. This includes developing guidelines and standards for food waste valorization processes, ensuring the safety and quality of products derived from food waste, and creating incentives for businesses and consumers to participate in circular bioeconomy initiatives.

Public awareness and engagement are essential for the successful implementation of circular bioeconomy practices. Future research should explore effective strategies for raising awareness about the benefits of food waste valorization and encouraging behavior change among consumers and businesses. This could involve educational campaigns, community-based projects, and collaborations with industry stakeholders to promote the adoption of circular bioeconomy principles. In conclusion, while significant progress has been made in the transformation of food wastes into renewable food resources, there is still much more work to do. By addressing the challenges and pursuing the future research orientations outlined above, we can unlock the full potential of the circular bioeconomy and contribute to a more sustainable and resilient food system. Through continued innovation, collaboration, and commitment to sustainability, we can turn food waste from an environmental burden into a valuable resource, paving the way for a greener and more prosperous future.

## Figures and Tables

**Table 1:** Provides a concise overview of various food wastes, their valorization processes resulting renewable food resources.

Food Waste	Valorization Process	Renewable Food Resource	Benefits	References
Fruit and Vegetable Peels	Fermentation and Drying	Dietary Fiber Supplements	Increases dietary fiber intake; reduces waste.	[33]
Spent Grain (Brewing By-Product)	Dehydration and Milling	Flour for Baking	High in protein and fiber; reduces by-product waste.	[34]
Whey (Dairy By-Product)	Protein Isolation and Purification	Protein Powders and Beverages	Provides a high-quality protein source.	[35]
Coffee Grounds	Extraction and Refinement	Coffee Oil for Cosmetics, Flour	Uses coffee grounds in cosmetics; adds fiber to food products.	[36]
Bread Waste	Fermentation and Enzymatic Hydrolysis	Beer and Spirits	Creates alcoholic beverages; valorizes stale bread.	[37]
Fish By-Products (Bones, Skin)	Hydrolysis and Purification	Fish Oil Supplements, Gelatin	Rich in omega-3 fatty acids; uses typically discarded parts.	[38]
Citrus Peels	Extraction and Refinement	Essential Oils and Pectin	Produces flavoring agents and natural gelling agents.	[39]
Tomato Pomace	Drying and Grinding	Tomato Powder	Used as a flavoring or coloring agent; high in antioxidants.	[40]
Spent Yeast (Brewing and Baking)	Autolysis and Fermentation	Yeast Extract	Rich in B vitamins and used as a flavor enhancer.	[41]
Potato Peels	Starch Extraction	Potato Starch	Used as a thickening agent; reduces waste.	[42]

## Data Availability

No new data were created or analyzed in this study. Data sharing is not applicable to this article.
